# Supplementary material for: Hesitancy towards COVID-19 vaccination: The role of personality traits, anti-vaccine attitudes and illness perception
Source: PLOS Glob Public Health. 2022 Dec 28;2(12):e0001435. doi: 10.1371/journal.pgph.0001435 (PMC10021484; doi:10.1371/journal.pgph.0001435)
Supplement: S1 File — Table A. Inter-correlations between Illness beliefs, Big five personality types, components of vaccine attitudes, combined anti-vaccine attitudes and vaccine hesitancy. Table B. Summary of hierarchical regression results. (DOCX) [file pgph.0001435.s001.docx]

**Hesitancy towards COVID-19 vaccination: The role of personality traits, anti-vaccine attitudes and illness perception**

S1 File

# **Table A. Inter-correlations between Illness beliefs, Big five personality types, components of vaccine attitudes, combined anti-vaccine attitudes and vaccine hesitancy.**

| Variables | 1 | 2 | 3 | 4 | 5 | 6 | 7 | 8 | 9 | 10 | 11 | 12 | 13 | 14 | 15 | 16 | 17 | 18 | 19 | 20 |
| --- | --- | --- | --- | --- | --- | --- | --- | --- | --- | --- | --- | --- | --- | --- | --- | --- | --- | --- | --- | --- |
| 1. Consequences | - |  |  |  |  |  |  |  |  |  |  |  |  |  |  |  |  |  |  |  |
| 1. Timeline | .32** | - |  |  |  |  |  |  |  |  |  |  |  |  |  |  |  |  |  |  |
| 1. Identity | .20** | .20** | - |  |  |  |  |  |  |  |  |  |  |  |  |  |  |  |  |  |
| 1. Concern | .36** | .27** | .22** | - |  |  |  |  |  |  |  |  |  |  |  |  |  |  |  |  |
| 1. Emotional Representation | .37** | .25** | .23** | .49** | - |  |  |  |  |  |  |  |  |  |  |  |  |  |  |  |
| 1. Personal Control | -.05 | .04 | -.08 | -.01 | .01 | - |  |  |  |  |  |  |  |  |  |  |  |  |  |  |
| 1. Treatment Control | -.21** | -.21** | -.16** | -.30** | -.21** | .22** | - |  |  |  |  |  |  |  |  |  |  |  |  |  |
| 1. Coherence | -.16** | -.15** | -.11** | -.23 | -.06 | .30** | .24** | - |  |  |  |  |  |  |  |  |  |  |  |  |
| 1. Extraversion | .01 | -.03 | .02 | -.04 | -.05 | -.07 | -.01 | -.08 | - |  |  |  |  |  |  |  |  |  |  |  |
| 1. Agreeableness | -.15** | -.17** | -.19** | -.04 | -.11** | -.06 | .03 | -.09 | .05 | - |  |  |  |  |  |  |  |  |  |  |
| 1. Conscientiousness | -.08 | -.11** | -.19** | -.01 | -.09 | -.06 | .06 | -.15** | .14** | .25** | - |  |  |  |  |  |  |  |  |  |
| 1. Neuroticism | .06 | .09 | .07 | .06 | .19** | .16** | -.03 | .09 | -.17** | -.24** | -.25** | - |  |  |  |  |  |  |  |  |
| 1. Openness | .03 | .04 | -.03 | -.01 | .10 | -.07 | .03 | -.06 | .06 | .05 | .15** | .07 | - |  |  |  |  |  |  |  |
| 1. Experience with COVID-19 | -.26** | -.22** | -.28** | -.23** | -.21** | -.09 | .19** | .10* | .05 | .38** | .11* | -.06 | .01 | - |  |  |  |  |  |  |
| 1. Vaccine mistrust | -.22** | -.22** | -.08 | -.34** | -.17** | -.06 | .34** | .08 | .02 | .11* | .05 | -.04 | .03 | .30** | - |  |  |  |  |  |
| 1. Future Effects | .00 | .03 | -.02 | -.04 | .06 | -.06 | .10* | -.07 | -.01 | .06 | .08 | -.02 | .06 | .12** | .29** | - |  |  |  |  |
| 1. Profiteering | -.11** | -.11** | -.04 | -.14** | .05 | -.05 | .24** | .06 | .03 | .04 | .10 | .04 | .03 | .22** | .42** | .48** | - |  |  |  |
| 1. Natural immunity | -.14** | -.20** | -.03 | -.18 | -.07 | -.06 | .23 | .03 | .00 | -.01 | .15 | .01 | .04 | .15 | .35** | .41** | .56** | - |  |  |
| 1. Vaccination Attitudes(total) | -.17** | -.18** | -.06 | -.24** | -.05 | -.08 | .31** | .04 | .02 | .07 | .12** | .00 | .05 | .27** | .72** | .69** | .82** | .76** | - |  |
| 1. Vaccine Hesitancy | -.26** | -.25** | -.09 | -.39 | -.26** | -.08 | .35** | .10** | -.04 | .09* | .05 | -.05 | .00 | .43** | .69** | .29** | .40** | .32** | .59** | - |

*Correlation is significant at the 0.01 level (2-tailed)

**Correlation is significant at the 0.05 level (2-tailed)*

# **Table B. Summary of hierarchical regression results**

| **Variable** |  | **Model 1** |  |  |  | **Model 2** |  |  |  | **Model 3** |  |  |  | **Model 4** |  |  |  | **Model 5** |  |
| --- | --- | --- | --- | --- | --- | --- | --- | --- | --- | --- | --- | --- | --- | --- | --- | --- | --- | --- | --- |
|  | B | SE B | β |  | B | SE B | β |  | B | SE B | β |  | B | SE B | β |  | B | SE B | β |
| **Sociocultural Variables** |  |  |  |  |  |  |  |  |  |  |  |  |  |  |  |  |  |  |  |
| Gender=Female | .19 | .71 | .01 |  | .40 | .72 | .03 |  | .37 | .66 | .02 |  | .68 | .61 | .04 |  | .13 | .50 | .01 |
| Ages <= 23 (vs 31+) | .94 | 1.48 | .06 |  | .87 | 1.50 | .06 |  | .28 | 1.36 | .02 |  | -.56 | 1.25 | -.04 |  | -.81 | -4.00 | -.05 |
| Ages 24 – 30 (vs 31+) | -.58 | 1.35 | -.04 |  | -.64 | 1.36 | -.04 |  | -.32 | 1.24 | -.02 |  | -.42 | 1.13 | -.03 |  | -.54 | .91 | -.04 |
| Akan (vs other ethnicities) | -.52 | .00 | -.03 |  | -.46 | -2.00 | -.03 |  | -.23 | .91 | -.02 |  | -.43 | .83 | -.03 |  | -.60 | .67 | -.04 |
| Ga-Adangbe (vs other ethnicities) | 1.25 | 1.33 | .05 |  | 1.21 | 1.33 | .05 |  | .75 | 1.21 | .03 |  | .19 | 1.11 | .01 |  | .62 | .89 | .03 |
| Ewe (vs other ethnicities) | 1.69 | 1.26 | .08 |  | 1.55 | 1.26 | .08 |  | 1.46 | 1.15 | .07 |  | 1.16 | 1.05 | .06 |  | .85 | .85 | .04 |
| Single (vs. widowed/divorced) | -7.37 | 3.67 | **-.33*** |  | -7.64 | 3.68 | **-.34*** |  | -7.59 | 3.35 | **-.34*** |  | -5.94 | 3.09 | -.26 |  | -1.91 | 2.51 | -.09 |
| Married (vs. widowed/divorced) | -6.77 | 3.71 | -.29 |  | -7.06 | 3.72 | -.30 |  | -6.71 | 3.39 | **-.29*** |  | -5.55 | 3.10 | -.24 |  | -1.57 | 2.52 | -.07 |
| Tertiary (vs <=secondary) | -3.04 | 1.89 | -.08 |  | -3.24 | 1.90 | -.08 |  | -4.11 | 1.73 | **-.10*** |  | -3.94 | 1.58 | -.10 |  | -1.84 | 1.28 | -.05 |
| **Variable** |  | **Model 1** |  |  |  | **Model 2** |  |  |  | **Model 3** |  |  |  | **Model 4** |  |  |  | **Model 5** |  |
|  | B | SE B | β |  | B | SE B | β |  | B | SE B | β |  | B | SE B | β |  | B | SE B | β |
| Unemployed (vs. self-employed) | -.33 | 1.19 | -.02 |  | -.29 | 1.20 | -.02 |  | .43 | 1.10 | .03 |  | .76 | 1.02 | .05 |  | .53 | .82 | .03 |
| Formally employed (vs. self-employed) | -1.32 | 1.08 | -.09 |  | -1.23 | 1.09 | -.08 |  | .04 | .99 | .00 |  | .52 | .91 | .03 |  | .31 | .74 | .02 |
| Christian (vs others) | -.46 | 2.41 | -.02 |  | -1.19 | 2.43 | -.04 |  | -2.99 | 2.22 | -.10 |  | -1.52 | 2.06 | -.05 |  | -1.21 | 1.67 | -.04 |
| Muslim (vs others) | -1.85 | 3.01 | -.05 |  | -2.55 | 3.04 | -.07 |  | -4.18 | 2.77 | -.12 |  | -2.27 | 2.58 | -.06 |  | -3.03 | 2.08 | -.08 |
| Greater Accra (vs Central Region) | -2.08 | 1.63 | -.13 |  | -1.93 | 1.64 | -.12 |  | -.92 | 1.50 | -.06 |  | -1.13 | 1.37 | -.07 |  | .46 | 1.11 | .03 |
| 4 Northern regions ((vs Central Region) | .08 | 2.12 | .00 |  | .22 | 2.12 | .01 |  | .13 | 1.93 | .00 |  | .11 | 1.77 | .00 |  | 1.79 | 1.43 | .06 |
| Ashanti (vs Central Region) | -.68 | 1.96 | -.03 |  | -.70 | 1.97 | -.03 |  | -1.45 | 1.79 | -.06 |  | -1.55 | 1.64 | -.06 |  | -.18 | 1.33 | -.01 |
| Western (vs Central Region) | .00 | 2.31 | .00 |  | .27 | 2.32 | .01 |  | .70 | 2.11 | .02 |  | -1.22 | 1.93 | -.03 |  | .00 | 1.56 | .00 |
| Volta (vs Central Region) | -2.52 | 2.59 | -.06 |  | -2.12 | 2.62 | -.05 |  | -.99 | 2.39 | -.02 |  | -1.18 | 2.18 | -.03 |  | .28 | 1.76 | .01 |
| Eastern (vs Central Region) | -.34 | 2.29 | -.01 |  | -.35 | 2.31 | -.01 |  | -.20 | 2.11 | -.01 |  | -.39 | 1.92 | -.01 |  | 1.02 | 1.55 | .03 |
| **Psychological variables** |  |  |  |  |  |  |  |  |  |  |  |  |  |  |  |  |  |  |  |
| Openness |  |  |  |  | .02 | .24 | .01 |  | .06 | .22 | .01 |  | .01 | .20 | .00 |  | -.02 | .16 | .00 |
| **Variable** |  | **Model 1** |  |  |  | **Model 2** |  |  |  | **Model 3** |  |  |  | **Model 4** |  |  |  | **Model 5** |  |
|  | B | SE B | β |  | B | SE B | β |  | B | SE B | β |  | B | SE B | β |  | B | SE B | β |
| Extraversion |  |  |  |  | -.19 | .19 | -.05 |  | -.32 | .18 | -.08 |  | -.36 | .16 | -.09 |  | -.35 | .13 | **-.09**** |
| Agreeableness |  |  |  |  | .46 | .30 | .08 |  | -.51 | .29 | -.08 |  | -.36 | .27 | -.06 |  | -.39 | .22 | -.06 |
| neuroticism |  |  |  |  | -.20 | .22 | -.04 |  | -.25 | .20 | -.06 |  | -.08 | .19 | -.02 |  | -.04 | .15 | -.01 |
| Experience with COVID-19 |  |  |  |  |  |  |  |  | 2.83 | .29 | .**45**** |  | 2.18 | .28 | .**35**** |  | 1.45 | .23 | .**23**** |
| Consequences |  |  |  |  |  |  |  |  |  |  |  |  | -.13 | .12 | -.04 |  | -.03 | .10 | -.01 |
| Timeline |  |  |  |  |  |  |  |  |  |  |  |  | -.19 | .12 | -.07 |  | -.12 | .10 | -.04 |
| Identity |  |  |  |  |  |  |  |  |  |  |  |  | .34 | .13 | **.11**** |  | .23 | .11 | **.07*** |
| Concern |  |  |  |  |  |  |  |  |  |  |  |  | -.66 | .13 | **-.24**** |  | -.30 | .11 | **-.11**** |
| Emotional  representation |  |  |  |  |  |  |  |  |  |  |  |  | -.05 | .13 | -.02 |  | -.23 | .11 | **-.08*** |
| Personal Control |  |  |  |  |  |  |  |  |  |  |  |  | -.27 | .12 | **-.09*** |  | -.11 | .10 | -.04 |
| Treatment Control |  |  |  |  |  |  |  |  |  |  |  |  | .75 | .15 | **.22**** |  | .24 | .12 | .**07*** |
| Coherence |  |  |  |  |  |  |  |  |  |  |  |  | -.07 | .15 | -.02 |  | .01 | .12 | .00 |
| Vaccine Mistrust |  |  |  |  |  |  |  |  |  |  |  |  |  |  |  |  | 1.22 | .10 | **.49**** |
| Future effects |  |  |  |  |  |  |  |  |  |  |  |  |  |  |  |  | .31 | .12 | **.09**** |
| Profiteering |  |  |  |  |  |  |  |  |  |  |  |  |  |  |  |  | .26 | .12 | **.09*** |
| Natural immunity |  |  |  |  |  |  |  |  |  |  |  |  |  |  |  |  | -.10 | .12 | -.03 |
| R^2^ |  |  | .06 |  |  |  | .07 |  |  |  | .23 |  |  |  | .38 |  |  |  | .60 |
| R^2^ change |  |  |  |  |  |  | .01 |  |  |  | .16 |  |  |  | .14 |  |  |  | .23 |

NB. B- unstandardized Beta; SE, standard error; β-Standardized Beta. *p< .05, ** p<.001

Model 1: Sociodemographic variables

Model 2: Personality types

Model 3: Illness perception

Model 4: Anti-vaccine attitudes

Model 5: Total model

The figures in bold are statistically significant.
