# Supplementary material for: Hesitancy towards COVID-19 vaccination: The role of personality traits, anti-vaccine attitudes and illness perception
Source: PLOS Glob Public Health. 2022 Dec 28;2(12):e0001435. doi: 10.1371/journal.pgph.0001435 (PMC10021484; doi:10.1371/journal.pgph.0001435)
Supplement: Attachment — Submitted filename: Response to Reviewers.docx [file pgph.0001435.s002.docx]

University of Ghana

P.O. Box LG 75

Accra, Ghana

2^nd^ September, 2022

The Academic Editor

PLOS Global Public Health

Dear Madam,

Response to comments of Reviewers – “Hesitancy towards COVID-19 vaccination: The role of personality traits, anti-vaccine attitudes and illness perception”

Thank you for the review comments on the above journal article. The authors of this manuscript have reviewed the constructive comments made by the reviewers and have made the appropriate amendments to make the manuscript meet PLOS Global Public Health’s [publication criteria](https://journals.plos.org/globalpublichealth/s/criteria-for-publication) in terms of technicalities and soundness. I write on behalf of my co-authors, to submit the revised paper based on the comments and suggestions received.

All changes are seen in the manuscript with track changes document. Below is a point-by-point response to each of the comments.

Yours faithfully,

Nanteer-Oteng Eric

REVIEWER 1

**Introduction**

**Comment: 1**- This is a just reviewer-friendly request and not a substantial issue in the manuscript itself: there are no page numbers nor line numbers in the manuscript which makes it difficult to point out specific improvements with examples.
2- The introduction is too broad. It could be shortened and be more focused on the COVID-19 vaccine and the gaps that this study would cover.

**Response**: Page numbers have been introduced as well as line numbers. With regard to the introduction, it has been shortened and more information has been added to increase the focus on COVID-19. Thank you. (See pages 1-2)

**Methods:**

**Comment 1:** It is important to elaborate on the sampling method and it would be interesting to know how limiting one answer to one person was achieved using Google forms.

**Response:** This has been addressed on page 5. (See lines 129 to 131)

**Results**

**Comment 1:** Please consider re-formatting tables (including the ones in the appendices) as they are not reader-friendly and not well-organized. For example, Table.1: Different categories like: vaccination status, Employment status. etc need to be labelled. Also, no need to keep space and add many dashes for Mean and SD while they do not exist for many variables. Better to keep mean and SD under age variable and remove the extra columns.

**Response:** Table formatting has been amended. (See page 9 and 10)

**Comment 2:** It is a good idea to list the hypotheses and describe the results and analysis in line with them. I would suggest to put the associated table with each hypothesis in the result section. For example, hierarchal linear regression table could be added to H2 in the results instead of being an appendix.

**Response**: This has been amended. The table has been brought right after results. (See pages 12-14)

**Comment 3**: In table 2, it is not clear "statistically" how such a mild difference between vaccinated and unvaccinated groups would be highly significant. It would be beneficial to list the number of samples in each category to better demonstrate the result.

**Response**: The sample numbers have been added to improve clarity. Thank you. (See page 11)

**Comment 4**: It would be beneficial for the reader to get information on the level of multicollinearity particularly with variables like vaccine attitude and COVID-19 vaccine hesitancy. And such results should be explained and justified through the hierarchy linear regression.

**Response**. Regression assumptions were accounted for in the analysis and this has been stated on Page 11, from lines 222-224. Also, subscales were used as predictors and none of the variables exceeded .6. Again, the values for the Pearson correlation can be seen in the correlation table in the appendix.

**Comment 5**: - The tables labels are not being referred to properly in the result descriptions which is confusing (table no...)

**Response**: This has been rectified with track changes made. Thank you.

**Comment 6**: It would be great if the selection of predictors for each model in the hierarchy linear regression is being explained.

**Response**: The predictors were selected as grouped independent variables to ascertain the influence of each independent variable as a cluster on the dependent variable. Thank you. (See Page 11, lines 224-231).

**Comment 7**: In the moderation effect simple slope analysis; what defines high, moderate or low COVID-19 vaccine hesitancy level? Are they subjective cut-offs? As explained in the scale used, the scores indicate comparativeness rather than categorization or leveling.

**Response**: In using Process Macro, an arbitrary set of means are created by the software to indicate the various levels at which the moderation takes place, hence, the classification at high, mid and low.

**Discussion**

**Comment 1**- The results of this study cannot be generalized due to the bias in participants’ socio-demographic backgrounds.

**Response**: This has been included in the limitations (See page 19, lines 337 to 340). Thank you.

**Comment 2** – The level of participants' education and consequently COVID-19 and COVID-19 vaccination knowledge might have an influence on the reporting toward more socially desirable answers.

**Response**: This has been addressed on page 19, lines 336 and 337. Thank you.

REVIEWER 2

Reviewer 2 had no comments that required any changes to be made and we are grateful for the reviewer’s constructive input and time taken to review our manuscript.

Thank you.
