# Supplementary material for: Hesitancy towards COVID-19 vaccination: The role of personality traits, anti-vaccine attitudes and illness perception
Source: PLOS Glob Public Health. 2022 Dec 28;2(12):e0001435. doi: 10.1371/journal.pgph.0001435 (PMC10021484; doi:10.1371/journal.pgph.0001435)
Supplement: Attachment — Submitted filename: Response to Reviewers.docx [file pgph.0001435.s003.docx]

University of Ghana

P.O. Box LG 75

Accra, Ghana

5^th^ November 2022

The Academic Editor

PLOS Global Public Health

Dear Madam,

Response to comments of Reviewer – “Hesitancy towards COVID-19 vaccination: The role of personality traits, anti-vaccine attitudes and illness perception”

Thank you for the review comments on the above journal article. The authors of this manuscript have reviewed the constructive comments made by the reviewers and have made the appropriate amendments to make the manuscript meet PLOS Global Public Health’s [publication criteria](https://journals.plos.org/globalpublichealth/s/criteria-for-publication) in terms of technicalities and soundness. I write on behalf of my co-authors, to submit the revised paper based on the comments and suggestions received.

All changes are seen in the manuscript with the track changes document. Below is a point-by-point response to each of the comments.

Yours faithfully,

Nanteer-Oteng Eric

REVIEWER 1

**Comment: 1**- The multicollinearity between COVID-vaccination hesitancy and vaccine attitude is still un explain in the manuscript. These two factors were input into one model which might jeopardize the result. Please refer to S1 (appendix)

**Response**: There was no multicollinearity among variables. The correlation between vaccine hesitancy and total vaccine attitudes was r=.59. The subscales used in the regression model correlated adequately with the dependent variable with only one variable correlating at r=.69. This has been better established in the manuscript (see lines 210-214). According to Julie Pallant, a correlation of above .7 is problematic (Pallant, 2016, p.178). Also, if the standard errors are small and the precision for the variables of interest is high, multicollinearity can be waived. The value maintained in this study was .69.

**Comment 2:** The manuscript still needs revision for typos.

**Response:** Manuscript has been reviewed and typos corrected as seen in the track changes. Thank you.

**Comment 3**- No need to add "hypothesis..." to the subtitles and tables. The current structure helps the reader to understand that it is the hypothesis.

**Response**: This has been edited as suggested. (See lines 184, 203, 230, and 234). Thank you.

We hope your concerns regarding the manuscript have been adequately addressed. Thank you for your time spent reviewing our manuscript.
