# Supplementary material for: Hesitancy towards COVID-19 vaccination: The role of personality traits, anti-vaccine attitudes and illness perception
Source: PLOS Glob Public Health. 2022 Dec 28;2(12):e0001435. doi: 10.1371/journal.pgph.0001435 (PMC10021484; doi:10.1371/journal.pgph.0001435)
Supplement: Attachment — Submitted filename: Response to Reviewers.docx [file pgph.0001435.s004.docx]

University of Ghana

P.O. Box LG 75

Accra, Ghana

5^th^ November 2022

The Academic Editor

PLOS Global Public Health

Dear Madam,

Response to comments of Reviewer – “Hesitancy towards COVID-19 vaccination: The role of personality traits, anti-vaccine attitudes and illness perception”

Thank you for the review comments on the above journal article. The authors of this manuscript have reviewed the constructive comments made by the reviewers and have made the appropriate amendments to make the manuscript meet PLOS Global Public Health’s [publication criteria](https://journals.plos.org/globalpublichealth/s/criteria-for-publication) in terms of technicalities and soundness. I write on behalf of my co-authors, to submit the revised paper based on the comments and suggestions received.

All changes are seen in the manuscript with the track changes document. Below is a point-by-point response to each of the comments.

Yours faithfully,

Nanteer-Oteng Eric

Editor comments.

1: Can you write a more detailed caption for Figure 1 and include more of a description on the legend? As is I'm not sure what the lines mean.

Response: An explanation for lines in the graph has been provided as requested and a legend added to the manuscript. Thank you.
